# Novel role of the LPS core glycosyltransferase WapH for cold adaptation in the Antarctic bacterium *Pseudomonas extremaustralis*

**DOI:** 10.1371/journal.pone.0192559

**Published:** 2018-02-07

**Authors:** Florencia C. Benforte, Maria A. Colonnella, Martiniano M. Ricardi, Esmeralda C. Solar Venero, Leonardo Lizarraga, Nancy I. López, Paula M. Tribelli

**Affiliations:** 1 Departamento de Química Biológica, Facultad de Ciencias Exactas y Naturales, Universidad de Buenos Aires, Buenos Aires, Argentina; 2 Centro de Investigaciones en Bionanociencias, CONICET, Buenos Aires, Argentina; 3 Instituto de Fisiología, Biología Molecular y Neurociencias, Facultad de Ciencias Exactas y Naturales, Universidad de Buenos Aires, Buenos Aires, Argentina; 4 IQUIBICEN, CONICET, Buenos Aires, Argentina; Laurentian University, CANADA

## Abstract

Psychrotroph microorganisms have developed cellular mechanisms to cope with cold stress. Cell envelopes are key components for bacterial survival. Outer membrane is a constituent of Gram negative bacterial envelopes, consisting of several components, such as lipopolysaccharides (LPS). In this work we investigated the relevance of envelope characteristics for cold adaptation in the Antarctic bacterium *Pseudomonas extremaustralis* by analyzing a mini Tn5 *wapH* mutant strain, encoding a core LPS glycosyltransferase. Our results showed that *wapH* strain is impaired to grow under low temperature but not for cold survival. The mutation in *wapH*, provoked a strong aggregative phenotype and modifications of envelope nanomechanical properties such as lower flexibility and higher turgor pressure, cell permeability and surface area to volume ratio (S/V). Changes in these characteristics were also observed in the wild type strain grown at different temperatures, showing higher cell flexibility but lower turgor pressure under cold conditions. Cold shock experiments indicated that an acclimation period in the wild type is necessary for cell flexibility and S/V ratio adjustments. Alteration in cell-cell interaction capabilities was observed in *wapH* strain. Mixed cells of wild type and *wapH* strains, as well as those of the wild type strain grown at different temperatures, showed a mosaic pattern of aggregation. These results indicate that *wapH* mutation provoked marked envelope alterations showing that LPS core conservation appears as a novel essential feature for active growth under cold conditions.

## Introduction

The 80% of earth surface, in terrestrial and aquatic environments, presents temperatures around or below the 15°C [1]. Temperature is a key factor for bacterial survival and growth. Although most of microorganisms could suffer transient changes of temperature, psychrophiles and psychrotolerant microorganisms have developed different adaptation strategies for growth under low temperatures[2]. Exposure to cold and ice provokes different effects in cellular components and some of the adaptation mechanisms have been studied in psychrotolerant microorganisms particularly regarding oxidative stress resistance, cold shock protein expression and metabolic shift [3–5]. Cellular integrity depends of cell envelope, in Gram negative bacteria the envelope consists of an inner membrane (IM) and the outer membrane (OM), separated by the periplasmic space containing a thin peptidoglycan layer [6]. The OM of Gram-negative bacteria is formed by phospholipids, proteins and lipopolysaccharides (LPS). Outer membrane characteristics could be modified during different stress conditions such as exposure to metal, hypersalinity and antibiotics [7–10]. LPS is the most important compound of the OM and contains Lipid A and an oligosaccharide component [6]. The oligosaccharide component is composed by a variable portion, the O-antigen and a core region (in which the O-antigen is attached). The core is constituted by an internal portion containing 3-deoxy-D-manno-oct-2-ulosonic acid (Kdo) and heptose residues and an external portion that includes glucose (II) residue [6]. During OM biogenesis main components such as LPS and proteins should be synthesized, exported and anchored actively and several enzymes are involved in the biosynthesis of LPS, among them the glycosyltransferase *wapH* catalyzes the addition of the glucose (II) residue to the external portion of LPS core [11]. This is a key residue for the formation of a short LPS glycoform 1 [12].

*Pseudomonas extremaustralis* is an Antarctic isolate able to grow under low temperatures, that shows high stress resistance and high amounts of polyhydroxybutyrate (PHB) [13]. In this bacterium, PHB accumulation is essential for cold growth and freezing survival, additionally contributes to develop a planktonic life style at cold conditions [14,15]. In comparison with other *Pseudomonas* species such as *P*. *putida* KT2440, *P*. *aeruginosa* PAO1 and *P*. *protegens* Pf-5, *P*.*extremaustralis* grows faster and reaches higher biomass yields at low temperatures [16]. Additionally, its metabolism at cold conditions has been studied in RNA-seq experiments describing an essential role of ethanol oxidation pathway [5].

The effect of low temperatures on bacterial envelope has been studied principally in Gram positive species focused on changes in the lipid characteristics but there is little information about LPS role on cold adaptation in psychrotolerant bacteria [1,17]. In this work, we analyzed the impact of a mutation in the LPS glycosyltranferase, *wapH* gene on cold growth and survival as well as the nanomechanical properties of the envelope using atomic force microscopy. We also analyzed the changes occurred in envelope characteristics in *P*. *extremaustralis* at low temperatures. We showed that LPS is a key component for low temperature adaptation in *P*. *extremaustralis*.

## Materials and methods

### Strains and culture conditions

*P*. *extremaustralis* [13] its derivate *wapH* mutant and complemented strain were used throughout the experiments. Cultures were grown in LB medium supplemented with 0.25% sodium octanoate (for PHA accumulation [18]) and incubated under aerobic conditions (200 rpm) at 30°C or 8°C. The *wapH* mutant strain was identified during the construction of a transposon mutant library of *P*. *extremaustralis* using pUTmini-Tn5 [ΩTc] and *E*. *coli* S17-1 as donor strain in a conjugation assay [19]. This mutant strain, unable to grow under cold conditions, was selected by plating transconjugants on LB agar supplemented with sodium octanoate and tetracycline (10 μg/ ml) both at 8°C and 30°C. To identify interrupted genes, a two-step PCR strategy was performed as described before using the followed oligonucleotidesARB1 (5’GGCCACGCGTCGACTAGTCAN NNNNGATAT 3’) and TN1 (5’GCCCGGCAGTACCGGCATAA 3’) for the first step and ARB2 (5’GGCCACGCGTCGACTAGTAC 3’) and TN2 (5’GGGTGACGGTGCCGAGGATG3’) for the second step [20]. The final PCR product was purified and sequenced (Macrogen, Korea). This strategy was used before to complement the study of *P*.*extremaustralis* global transcriptome under cold conditions [5]. For complementation experiments, the *wapH* gene with 300 bp upstream from the ATG was obtained by PCR using GlicUp (5’ TGATCAAGGTCGACCATCCC3’) and Gliclow (5’GGACAGACGCTCGATACC3’) oligonucleotides, was cloned into pBBR1MSC-5 [21] and introduced into the corresponding mutant strain by conjugation.

### Survival and growth experiments

For growth curves experiments pre-inoculum was prepared as described above and was used to inoculate cultures of LB supplemented with sodium octanoate with an initial OD_600nm_ = 0.05 and incubated at 8°C for 72 h and at 30°C for 30 h. OD_600nm_ was measured through time. In order to examine bacterial survival at 8°C, exponentially growing cells (OD_600 nm_ = 0.5) at 30°C were downshifted to 8°C and incubated for 16 h or 42 h. Viable bacterial number was measured by colony counts on LB plates before and after incubation at 8°C [14]. The number of bacteria before cold exposure was considered as one hundred percent and survival percentage was calculated as (CFU/ml T_16h or 42h_/CFU/ml T_0_) *100.

### LPS analysis

LPS samples were obtained from cultures using EDTA extraction [22]. Briefly cultures were first diluted to an OD_600nm=_4 to equalize cell numbers. The cultures were centrifuged at 4°C during 10 m at 7000 rpm. Pellets resuspended in 250 mM EDTA and the suspension was vortexed vigorously for 5 s and incubated at 37°C for 30 min. Proteinase K was added and samples were incubated during 1 h at 60°C. The supernatant was recovered for analysis after centrifugation at 10 000 X *g* for 5 min. Kdo was measured in samples as described before [23] using Kdo (Sigma) as standard. Same amount of Kdo was used for all samples to examine LPS using 12% polyacrylamide gel electrophoresis (PAGE) and silver staining [24].

### Stress experiments

For oxidative and SDS stress experiments cultures were incubated overnight at 30°C or for 72h at 8°C. Sensitivity to H_2_O_2_ in stationary cultures at 30°C was evaluated as described previously using sterile Whatman N°. 1 filter discs (6 mm) impregnated with 8 μl of 30% v v^-1^ H_2_O_2_ (Merck) [14]. Inhibition growth zone was measured after incubation for 24 h at 30°C. SDS sensitivity test was performed as described in Spiers and Rainey [22].

### Autoaggregation experiments

Autoaggregation and settling assays were performed as described before [25] with modifications. Briefly, overnight cultures were diluted with fresh media and the OD_600nm_ was adjusted to 3 to ensure the same number of cells of each strain. One ml aliquot was incubated at room temperature without agitation during different times and 200 μl from the top of the culture was taken (non- settled cells) while the rest of the culture was vigorously vortexed. The OD_600nm_ of both samples was determined. Aggregation (%) was determined for each time as follows: (OD vortexed-OD non-settled)/ODvortexed* 100). Other approach for the evaluation of cell-cell interactions was performed by analyzing mixed aggregates formation. One strain carrying the pSEVA237R_Pem7 (mCherry) was mixed with the wild type strain, the *wapH* mutant or the complemented strain followed by the procedure described above. In all cases cell suspension was adjusted at OD_600nm_ of 3 to ensure the same number of cells. After cell suspension was mixed in a 1:1 proportion in 1 ml as final volume. Fluoresce was measured using a fluorimeter (Optima FluoroStar).

### Confocal microscopy

Additionally, mixed aggregates were visualized using confocal microscopy. For aggregate visualization, strains carrying pSEVA237R_Pem7 or pBBRR1MSC-2 GFP [26] under a constitutive promoter were cultured overnight at 30°C andOD_600nm_ was adjusted to 0.8 for all strains. Then 1 ml aliquot of single strain or strains carrying different fluorescent protein were mixed in a 1:1 proportion and settled for 15 min. Aggregated cells were taken from the bottom of the Eppendorf tube and mounted in a slide with a cover glass and immediately observed in a confocal microscope. Three independent experiments were carried out with three replicates each one. Images were acquired in an Olympus FV300 confocal microscope (Olympus Latin America) with a 100x 1.44 N.A. oil immersion objective. For excitation, we used 488 and 546 nm lasers for GFP and mCherry respectively. Emission filters were 510–530 nm for GFP and 660 long pass filter nm for mCherry. 1024x1024 images were acquired in slow sweeping mode (9,75 seg/image) with a confocal aperture size of 3. Gaining, Offset and PMT were set to avoid crosstalk of both channels. Image adjustments were performed using ImageJ software.

### Bacteria sample preparation for atomic force microscopy (AFM) measurements

Polyethylenimine (PEI) coated glass slides were used to immobilize bacteria [27]. Briefly, glass pieces were prepared by exposing cleaned glasses for 30 min with PEI 20%. Then, glasses were rigorously rinsed with Mili-Q water and dried with nitrogen. Bacteria were immobilized by depositing a drop of bacterial culture suspension (DO_600nm_ of 0.5) onto the PEI coated glasses for 20 min at room temperature to allow cells to adhere to PEI. Then, bacteria-coated glasses were rinsed with Mili-Q water and they were covered with a diluted LB drop of 30 μl.

### Atomic force microscopy measurements

All AFM measurements on live bacteria were carried out in diluted LB at room temperature using a MultiMode 8 with a Nanoscope V controler, Bruker. Silicon nitride cantilevers were purchased from BrukerAFM Probes (MLCT, Santa Barbara, CA) with a nominal spring constant of 0.03 N/m. Cantilever spring constants were calibrated using the thermal tune function contained in Nanoscope 9.1 software. The photodetector sensitivity was calibrated on a PEI-coated surface using, the slope of the constant compliance region of the force curves obtained on the PEI-coated glasses. The slope was used to convert the cantilever deflection (D) in millivolts to nanometers. The cantilever deflection was then converted into a force (F) according to F = k × D, where k is the force constant of the cantilever. Force measurements were made by positioning the tip at different position along the apex of the surface of individual cells. Force curves were acquired at a loading rate of 2 μm s^-1^ using a trigger of 6 nN. Measurements were performed in contact mode, to ensure force profiles were representative of cell population, force curves were taken on at least 10 different points along the apex of an individual cell. For each cell type this was done for at least 10 cells from different separate sample preparations thereby providing approximately 300–500 force profiles for each culture conditions. This methodology allows obtaining information representative of bacteria nanomechanical properties. Cell surface, cell volume and cell length were determined from the images using Gwyddion software [28]. Polynomial background, projected area and volume from zero were used.

### Force curves analysis

The force-indentation curves were determined from the raw force curves using the methodology described in Touhami et al. [29]. Briefly, the indentation was calculated by subtracting the cantilever deflection on the bacterium from the cantilever deflection on the substrate. The force-indentation profiles exhibited two regimes (S1A Fig): i) a nonlinear regime for small applied loading forces (0 to 2 nN) and resulting small indentations, and ii) a linear regime upon further increase in the loading force applied (2 to 4nN) by the AFM tip over bacterium [30,31]. The slope of the linear regime of the force-indentation curve at high loading (2 to 6nN), it is well established that is related to the turgor pressure that counteracts the compression of the bacterium’s cytoplasm by the AFM tip[32]. This gradient is directly related to the bacterial spring constant, K_bacterium_, expressed by Hooke’s law as

$$F=K_{bacterium}\delta$$

Where F is the loading force and δ is the indentation force. K_bacterium_ was determined by each force indentation curve and it was mentioned its value is a measure of bacteria cytoplasmic turgor pressure, i.e. the pressure exerted by the cytoplasm on the plasma membrane [33]. The force indentation curve region at low loading forces which present a nonlinear behavior was fitting using the Hertz model [34]. A first approximation, the AFM tip can be considered as conical indenter. For an indenter of this geometry applying a loading force, to a flat, deformable surface, the relationship between F and the resulting indentation, δ, is given by

$$F=\frac{2}{\pi }tan\alpha \frac{E}{\left(1-\nu^{2}\right)}\delta^{2}$$

where ʋ is the Poisson’s ratio of the deformable sample (assumed to be 0.5 cells), α is the half-opening angle of a conical tip using a value of 18°, value given by the manufacturer. E is the sample’s Young’s module and is used as a fitting parameter. Young´s module allows obtaining a direct measure of the rigidity of the cell wall structure (capsule, inner and outer membrane and peptidoglycan layer)[35]. Representative image of *P*.*extremaustralis*´cell used for AFM measurement was shown in S1B Fig.

### qPCR Real Time experiments

Total RNA of *P*. *extremaustralis*, *wapH* and pSEVA*wapH* strain was extracted from 24 h cultures incubated at 30°C using the RNAeasy Mini Extraction Kit (Quiagen) following the manufacturer’s instructions followed by DNaseI treatment for 2 h. The RNA was quantified using NanoDrop 2000 (Thermo Fisher Scientific) and used for qPCR experiments. Expression was detected using the Power Sybr RNA to Ct 1 step kit (Termo Fisher Scientific) following manufacturer’s instructions with the following oligonucleotides: *cprX* 5′ CGGTGAGGGTGAATTCCTGT 3′ and 5′ ATCCTCGGCCTTGAATTGGG 3′, *wapH*. 5′CAGTTCTGCCACGGCTATGA ′3 and 5′ GGATGGCCTTGGAGCTGAAT′3; *mig14* 5′GGCTCGGTGATTTTCCTCCA ′3 and ′5′CCAACGGTCCTTGTACTCCC ′3 and for PE143B_0104935 5′AATGGCCTGCGTTACCTCAA′3 and 5′ATGACCATCACCCGTTGCTT′3. The 16S rRNA gene using primers 5′GTAACTGCCCTTCCTCCCAA′3 and 5′AGGTAATGGCTACCAAGGC′3 was used as reference for normalization of expression levels of target genes in each condition. The cycling conditions were as follows: cDNA production 48°C during 30 min, for qPCR denaturation at 95°C for 5 min, 40 cycles at 95°C for 25 s, 60°C for 15 s, and 72°C for 15 s. Relative changes in the expression of individual genes was obtained using ΔΔCt method [36]. At least three independent cultures were analyzed for each condition. RT qPCR was performed using AriaMx3005 (Agilent).

## Results

### Initial characterization of the *wapH*::mini Tn5 strain

A clone incapable to develop colonies at 8°C was isolated during a transposon library screening of *P*.*extremaustralis*. The insertion site of mini Tn-5 was located within *wapH* gene (PE143B_0104925), encoding for a glycosyltransferase. In *P*.*aeruginosa* PAO1 WapH adds a glucose residue to the outer core of LPS that enables to form a short glycoform of LPS [12]. A complemented strain carrying only the *wapH* gene was constructed. Colony development at 8°C was observed for the complemented strain similarly to the wild type strain (S2A Fig), suggesting that *wapH* mutation was mainly the cause of the defective cold growth phenotype observed. The *wapH*::mini Tn5 was called *wapH* strain and the complemented strain was named /pSEVA *wapH* and both strains were used for further experiments.

LPS analysis in polyacrylamide gel electrophoresis was performed for the wild type, the *wapH* mutant and the pSEVA*wapH* strain grown at 30°C. The wild-type strain resolved into a typical heterogeneous LPS-banding pattern, with high-molecular weight O-antigen bands and low molecular weight bands (S2B Fig). Differences between the LPS pattern from the wild type and the mutant strain were found in both zones when the same amount LPS was loaded. In the mutant strain higher abundance of high-molecular weight bands was observed in comparison with the wild type strain. These bands correspond to large O-antigen (S2B Fig). On the other hand, low molecular weight zone was in lower abundance in the mutant strain that could correspond to the core zone or to a low molecular weight glycoform (S2B Fig). Complementation with the *wapH* gene restored the LPS wild type pattern (S2B Fig). This pattern can be explain due to the key role of WapH in the biosynthesis of low weight glycoforms described in *Pseudomonas* species (S2B Fig) [37].

### Cold growth is impaired in a *wapH* strain

**Fig 1:**
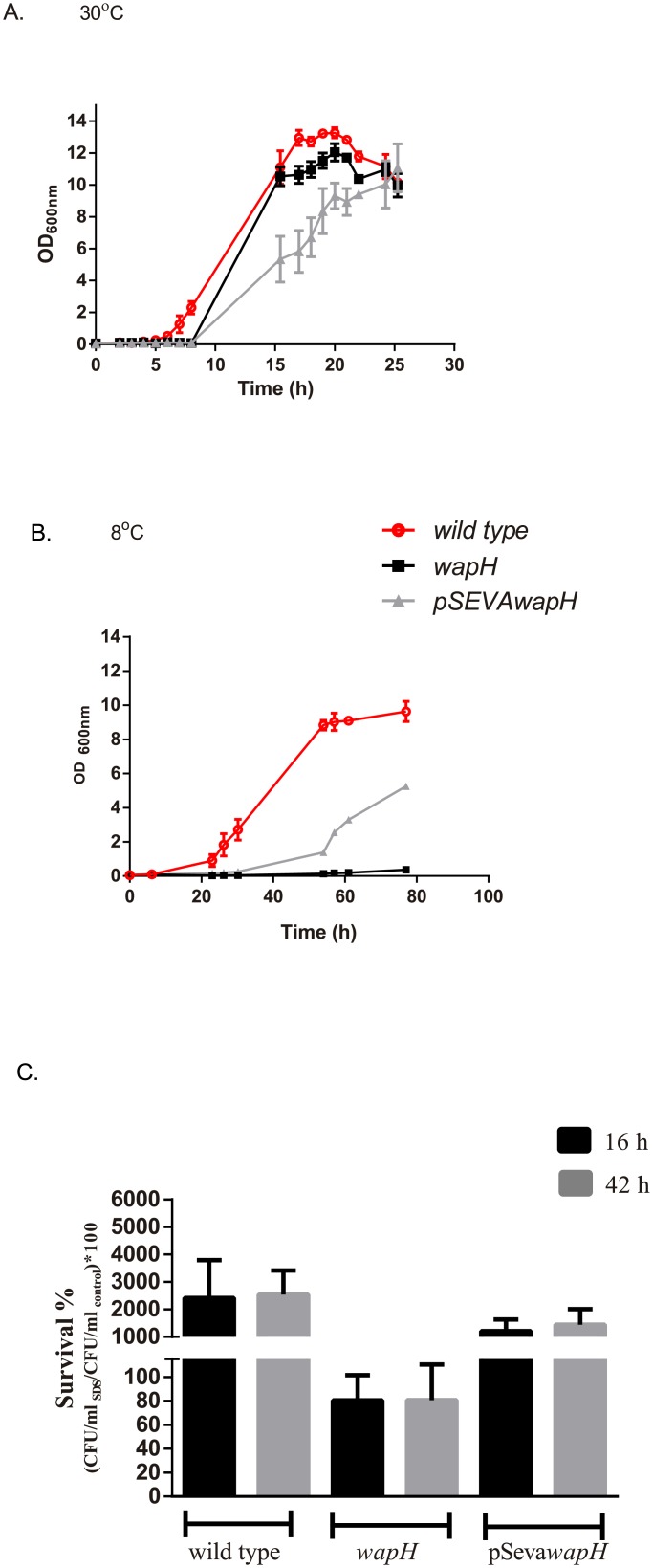
Impact of *wapH* mutation on cold growth and survival. Growth of the wild type, *wapH* and *pSEVAwapH* strains. A. Growth at 30°C. B. Growth at 8°C. C. Survival at low temperatures. Erlenmeyer were inoculated and incubated at 30°C until reached an OD_600nm_ of 0.5 and then incubated at 8°C. Samples were taken at 0, 16 and 42 h and CFU/ ml was determined. Survival was calculated as (CFU/ml _T = 16h or 42h_/CFU/ml _T = 0_) *100. Values represent mean ± SD of triplicate independent cultures.

*P*.*extremaustralis* and its mutant and complemented derivative strains were grown in sodium octanoate supplemented LB cultures at 8°C and 30°C under aerobic conditions. At 30°C all the strains reached around OD_600nm_ = 11.0 after 24 h culture (Fig 1A). Interestingly, only the cultures of *P*.*extremaustralis wapH* showed a thick biomass ring attached to the surface of the flask during early exponential growth phase which progressively unattached and integrated to planktonic cells (S2C Fig). At 8°C the *wapH* strain was unable to grow (and no evidence of attached biomass was observed) while the wild type strain and the complemented strain reached to 9.6±0.5 and 5.2±0.2 OD_600nm_ respectively (Fig 1B). Cold survival was also analyzed; the mutant strain was capable to survive after 16 and 42 h of low temperature exposure reaching 78±5 and 83±9% of viable cells, respectively (Fig 1C). In contrast, the wild type and the complemented strain could increase their cell number several times as was expected showing a survival percentage higher than 100% (for the wild type 2415±1380% and 2540±871% after 16 and 42 hours respectively and for the complemented strain 1209±429 and 1450±560 after 16 and 42 hours respectively) (Fig 1C). Our results suggest that the mutation in *wapH* was essential for growth under cold conditions.

### LPS core conservation is crucial for cell-cell interaction

**Fig 2:**
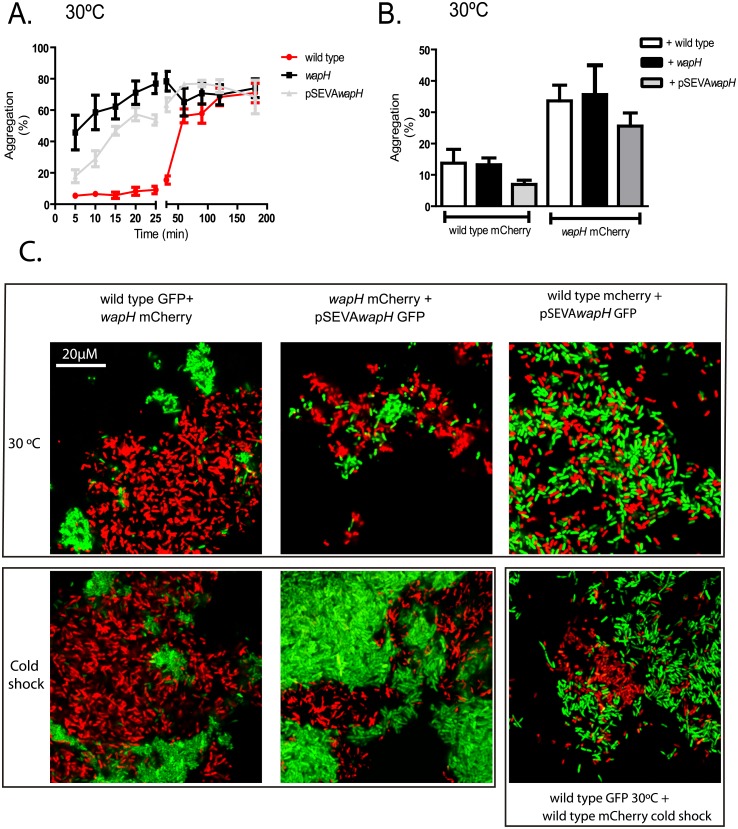
Aggregation assays. A. Aggregation assay at 30°C of the wild type (wt), *wapH* and complemented strain (pSEVA*wapH*). Values represent mean ± SD of 5 independent measurements. B. Aggregation assay with different strains expressing mCherry protein and mixed with an unmarked strain. Values represent media ± SD of 5 independent measurements. C. Microscopic visualization of mixed aggregates using cells grown at 30°C or from cold shock experiments. Strains expressing fluorescent proteins were mixed and settled for15 min. An aliquot from the bottom of the tube was taken and aggregates were observed in a confocal microscope using 1000X magnification. Representative images from triplicate independent experiments are shown.

Alteration in envelope could lead to changes in adhesion characteristics [38]. In contrast with the wild type strain; the *wapH* strain presented a tight biomass ring in Erlenmeyer cultures suggesting an aggregative phenotype. Settling capability (a common measure of cell to cell adhesion) was measured at 30°C. The *wapH* mutant strain presented 45 to 62% of autoaggregation after 5–15 min while the wild type strain only reached similar values after 30 and 120 min respectively (Fig 2A). The complemented strain showed a partial restoration of the wild type phenotype (Fig 2A). To analyze if the *wapH* strain could alter the wild type aggregation behavior, we performed a mixed aggregation assay in which one strain was carrying mCherry fluorescent protein while the other strain was unmarked. Strains were mixed in equal proportions and fluorescence measurements after 15 min (time in which only the mutant strain aggregated) (Fig 2A) were used to calculate the aggregation percentage. We showed that the wild type presented similar aggregation whether the added strain without mark was the mutant, the wild type or the complemented strain (Fig 2B). The same pattern was observed in the mutant strain since its aggregation behavior was the same in presence of all strains (Fig 2B).

A detailed study of aggregates using confocal microscopy was also performed using *wapH*, wild type or complemented strain expressing GFP or mCherry proteins. When the wild type and the mutant strain were mixed, aggregates with a mosaic pattern could be found (Fig 2C). The same pattern could be observed when the mutant strain was mixed with the complemented strain (Fig 2C). In contrast, mixed aggregates between the wild type and the complemented strain presented an undifferentiated mixed pattern in which both strains form part of the same aggregate (Fig 2C). In addition, cells exposed to a cold shock were used to perform the same experiments described above. Aggregates between the *wapH* and the wild type strain also presented a mosaic pattern, but the aggregates were bigger than those observed with cells grown at 30°C (Fig 2C), indicating that cold shock provokes an alteration in aggregation pattern. Interestingly; when aggregates were prepared mixing the wild type strain grown at 30°C and the wild type strain from cold shock experiments again a mosaic pattern was observed (Fig 2C), suggesting a change in the cell surface during cold shock.

The results showed that both the *wapH* mutation and the exposure to cold shock provoke an alteration on cell to cell interaction capabilities.

### Cell permeability is altered in the *wapH* strain

**Fig 3:**
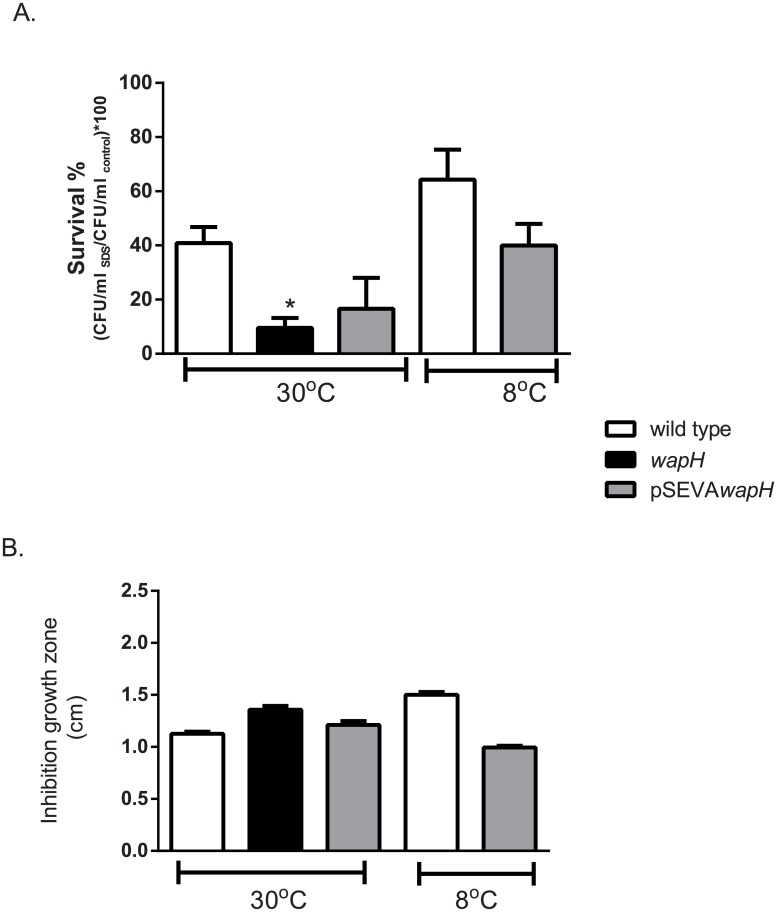
Cell permeability and polymyxin sensitivity assay. A. SDS sensitivity assay of the wild type (wt), *wapH* and complemented strain (pSEVA*wapH*). Cells were cultured at 30°C or 8°C and CFU/ml was determined in LB plates with and without SDS. B. Sensitivity to polymixin B of the wild type (wt), *wapH* and complemented strain (pSEVA*wapH*) was performed by using disk inhibition assay with cells cultured at 30°C or 8°C. Values represent media ± SD of triplicate independent measurements. * denotes significant differences (Mann Whitney Test).

To figure out if growth defects at cold conditions were part of a wider stress resistance defects; sensitivity to H_2_O_2_ and to gentamicin was measured by an inhibition growth assay. Similar values of the diameter of the zones of growth inhibition were obtained for all the strains, reaching 2.7±0.2 cm for the wild type; 3.1±0.4 for the *wapH* and 2.3±0.2cm for the complemented strain in the case of H_2_O_2_ and 3.5±0.2cm for the wild type, 3.6±0.1 cm for the mutant and 3.6±0.2 cm for the complemented strain in the case of gentamicin. However, when cell permeability was measured by SDS and polymixin B sensitivity assays differences were found. Cell count in plates with SDS (and without as control) showed that the *wapH* mutant strain presented significant differences with the wild type strain at 30°C (P<0.05Mann Whitney test, Fig 3A) while the complemented strain showed a restoration of the wild type phenotype (Fig 3A). Both, the wild type and the complemented strain at 8°C presented a higher survival to SDS although this difference was not significant (P>0.05 Mann Whitney test, Fig 3A). Sensitivity to polymixin B was higher for the mutant strain than the wild type in line with SDS survival results (Mann Whitney test P<0.05, Fig 3B). Interestingly, at 8°C the wild type strain also presented higher sensitivity to polymixin B than when grown at 30°C (Mann Whitney test P<0.05, Fig 3B) while the complemented strain presented a lower sensitivity at 8°C than at 30°C. The results showed that cell permeability was affected by both temperature and LPS conservation.

### LPS conservation is a key factor for elasticity of cell envelope structure and turgor pressure during cold adaptation

**Fig 4:**
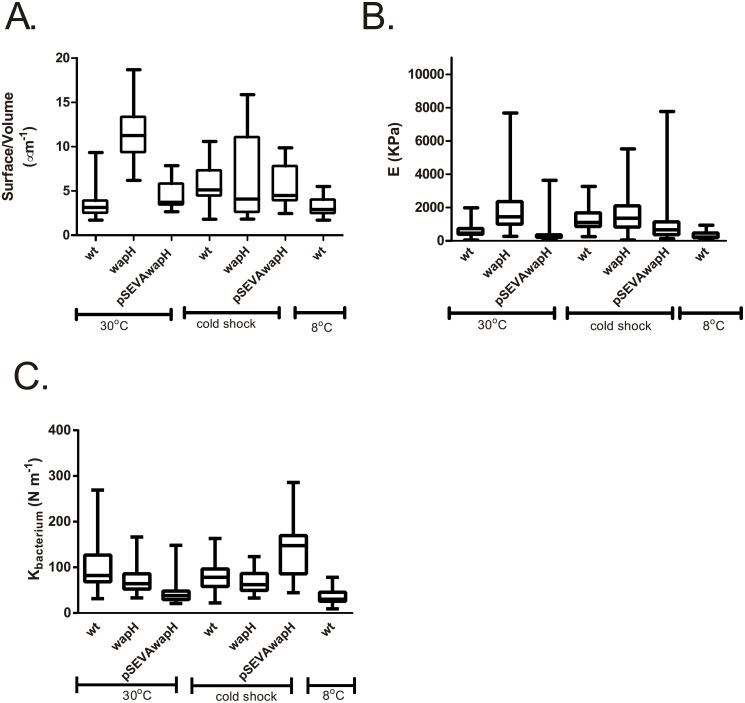
Nanomechanical determinations using atomic force microscopy (AFM) in live and hydrated cells. A Surface to Volume ratio (S/V) was determined using Gwyddion software. B. Cell elasticity determination at different culture conditions. Force-distance curves were obtained using MultiMode 8 with a Nanoscope V controller, Bruker in contact mode for at least 10 cells per condition in 10 different points along the major axis per triplicate. Adjustment to the Sneedon model was performed between 0 and 2 nN and the Young module was calculated. C. K_bacterium_ determinations as a measure of bacterial turgor pressure. Adjustment to the Hooke’s law was performed between 2 and 4 nN in the same curves Force-distance described above. Wild type (wt), *wapH* and complemented strain (pSEVA*wapH*).

Our results showed that *wapH* mutation impairs growth under low temperature conditions. To figure out the causes of these observations, nanomechanical measurements using an atomic force microscopy (AFM) were performed to determine the biophysical behavior of the envelope and other cell characteristics. Cell surface and volume were determined analyzing AFM images (S1 Fig) using Gwyddion software. Surface area to volume ratio (S/V) was calculated for all strains. The wild type strain maintained this parameter in a similar value at both, 30°C and 8°C confirming an adaptation of this bacterium to low temperatures and the homeostasis of this parameter (Fig 4A). In contrast, in cells belonging to cold shock experiments, the wild type strain showed a higher ratio than cells at both 8° and 30°C (P<0.05 Mann Whitney Test) suggesting an impact of low temperatures in these characteristics and that an acclimation period is necessary to reach a stable value (Fig 4A). At 30°C the mutant strain presented a higher S/V ratio than the wild type strain (P>0.05 Mann Whitney Test, Fig 4A), although both strains showed a similar S/V ratio in the cold shock assays(P>0.05 Mann Whitney Test, Fig 4A). Nanomechanical measurements showed that the mutation of *wapH* affects elasticity of cell envelope structure since the mutant strain presented a higher Young module (E) value in comparison with the wild type strain, suggesting a more “rigid” state in the *wapH* (P<0.05 Mann Whitney Test, Fig 4B). The complemented strain showed a restoration of the wild type phenotype (Fig 4B). Additionally, the wild type strain presented differences between temperatures, showing a lower E value at 8°C (P<0.05 Mann Whitney Test, Fig 4B). Cold shock experiments showed again an impact of temperature on the nanomechanical characteristics since the wild type presented rigid envelope similar to the mutant strain and different to that observed in the wild type grown at 30°C and 8°C. The K_bacterium_ values, a measure of bacteria cytoplasmic turgor pressure [33], were determined. The mutant strains showed a lower K_bacterium_ value than that observed for the wild type (P<0.05 Mann Whitney Test, Fig 4C) that could be related with the higher permeability observed. At 8°C a lower turgor pressure was observed for the wild type in comparison with 30°C (P<0.05 Mann Whitney Test, Fig 4C). Interestingly, in cold shock experiments wild type cells presented higher turgor pressure than at 30°C and 8°C (P<0.05 Mann Whitney Test, Fig 4C). On the other hand, the *wapH* cells from cold shock experiments presented similar K_bacterium_ values from the *wapH* at 30°C (P>0.05Mann Whitney Test, Fig 4C). Our results suggested that a prolonged period was necessary to adjust key cell parameters such as elasticity of cell envelope structures (inner and outer membrane and peptidoglycan layer) for low temperature adaptability and that LPS characteristic were important for flexibility adjustment necessary for development under cold conditions.

## Discussion

Low temperature affects several cellular processes and provokes physiological changes such as a decrease in membrane fluidity, a reduced efficiency of RNA transcription, translation and degradation as well as an increase in reactive oxygen species (ROS) [1,3]. Adequate cellular responses to these and other possible constraints contribute to the adaptation of organisms to cold conditions. *Pseudomonas* species are found in cold environments, are able to grow under low temperature and global transcription analysis at cold has been performed [5,39,40].*P*. *extremaustralis*, is an Antarctic psychrotolerant bacterium that presents a better behavior under cold conditions than other *Pseudomonas* species and constitutes a good model to find novel mechanisms to better understand bacterial development at low temperatures [5,16,18].

Cell envelopes integrity is essential for bacterial survival and modifications in this structure have been described under stress conditions, for example changes in outer membrane proteins have been found after exposure to metals or after hypersaline shock [7,9]. During cold growth changes in lipid content or lipid characteristics are displayed in order to counter the membrane stiffening [41–43]. LPS role was widely studied as toxin or in antibiotic resistance but less information is available about its relevance in bacterial adaptability to environmental conditions [43–45]. In this work we analyzed the impact of a mutation in the *wapH* gene encoding a glycosyltransferase enzyme of LPS core. Experiments performed in *P*.*syringae* Lz4w a pyschrotolerant bacterium show that LPS is phosphorylated in two heptose residues in a temperature dependent way [46]; but its role in bacterial survival was not studied. We demonstrated, using a mini Tn-5::*wapH* mutant strain, that a conserved LPS pattern was essential for growth under low temperatures but was not essential for growth at 30°C neither for survival to cold shock. Modification in the LPS core has been reported in *Escherichia coli*, in which colanic acid units are added to the LPS in response to environmental stimulus [47]. Interestingly, *P*.*extremaustralis* presents a colanic acid gene cluster probably acquired by horizontal transfer that could play a role during stress adaptation [16]. Moreover, *P*.*aeruginosa* PAO1 presents two different glycoforms, glycoform 2 that present the O-antigen and the glycoform1 in which the O-antigen is absent but a short lateral chain is present [12]. In glycoform 1 the lateral chain is added in the glucose II residue (added in the inner core by WapH) in the outer core [12]. *P*.*extremaustralis’* genome lacks the genes related with the addition of rhamnose, among them *migA*, showing differences with the widely studied *P*.*aeruginosa* PAO1 [38]. A different band pattern with higher abundance in the zone corresponding to long capped LPS was observed in the *wapH* strain in comparison with the wild type strain, suggesting that could be possible the existence of a second short glycoform similar to *P*.*aeruginosa* but without rhamnose. The mutation of *wapH* could lead to the absence of the low-weight glycoform and to the enrichment with high-molecular weight glycoform. A similar pattern was observed in *P*.*aeruginosa* PAO1 *wapH* strain, where a low molecular weight glycoform (glycoform 1) was affected and the bacterium still produce (in lower amounts) O-antigen and high molecular weight glycoforms [31]. By contrast, in *P*.*putida* KT2240 a different pattern was observed since a *wapH* mutant lacks of O-antigen [33]. These observations showed some differences in the balance of different LPS forms although the main components are well conserved among *Pseudomonas* species.

Analysis of the genomic region of *P*. *extremaustralis*, suggests that the transposon location may affects *mig14*, encoding a hypothetical protein (homologous to PA5002) and PE143B_0104935 that were located downstream the *wapH*. Homologous to these genes are also found in *P*. *aeruginosa* PAO1, but only a role in fluoroquinolone tolerance for PA5002 was described [31,32]. Expression experiment performed at 30°C showed that *wapH* expression was not detected in the mutant strain as was expected. In the complemented strain *wapH* gene was expressed in a lower amount than in the wild type (S2D Fig). However, the expression level was enough to complement the main observed phenotypes. Additionally, the expression of *mig14* and PE143B_0104935 was detected in this strain although was lower than the wild type (S2D Fig), while in the complemented strain the expression of these genes was slightly higher than in the mutant strain. We cannot rule out effects of *mig14* and PE143B_0104935 genes on aggregation phenotype since aggregation experiments showed a partial complementation. However, the pSEVA*wapH* strain carrying only *wapH* presented LPS pattern similar to the wild type strain and developed colonies at cold conditions, rescue grow in liquid cultures at 8°C, SDS survival, cell flexibility and S/V phenotype indicating that *wapH* is crucial for cold growth.

Aggregation is a survival strategy against different types of stress agents and LPS modifications lead to different phenotypes regarding biofilm and aggregation capabilities in *Pseudomonas* species [11,15,22,23,37,48–51]. In this work we demonstrated a key role of *wapH* in cellular aggregation. Moreover, temperature also impacts cell to cell interactions since wild type cells grown at 30°C form a mosaic aggregate with cold shock wild type cells, probably due to changes in cell envelope provoked by low temperatures. These results indicated the crucial role of LPS component even within unique species.

Nanomechanical measures in living-hydrated bacteria showed a more rigid state for the mutant strain and a lower turgor pressure, in line with higher cell permeability. Capsule lacking mutant strains in *Klebsiella pneumoniae* [35] show similar nanomechanical behavior than the *wapH* mutant of *P*.*extremaustralis* (which does not present capsule), suggesting that outer cell surface is a key structure for the determination of these characteristics. Moreover, exponential cultures of the wild type strain grown at 30°C shifted then to lower temperatures presented a higher Young module (E) than cultures grown at 8°C for 72 h, suggesting that a prolonged time is necessary for acclimation to low temperatures.

The accumulation of LPS and OMPs during stress conditions trigger an interaction between periplasmic LPS and the proteins RseB and RseA provoking the membrane stress response mediated by σE in *Escherichia coli* [52]. In early exponential cultures of *P*.*extremaustralis* at low temperature an overexpression of *cprX* gene was observed [5], this gene encoding an envelope stress sensor could represent early steps for cold adaptation. The expression of the *cprX* gene membrane stress sensor was tested in in this work in the wild type, *wapH* and pSEVA*wapH* strains at 30°C showing a similar expression level (1.3 and 0.9 folds in comparison with the wild type strain for the complemented and the *wapH* mutant strain respectively). Both observations suggested that *wapH* mutation did not induce membrane stress at 30°C or at least was not mediated by *cprX*, but is overexpressed in cold cultures in comparison to 30°C [5]. Cold-shock response and the later adaptation to grow or acclimation can be considered as distinct phases since imply different set of expressed genes [53]. Moreno and Rojo, 2014 [39] suggested that bacteria in natural environments are more likely to experience prolonged periods at low temperatures than rapid cold shocks. For the surface to volume ratio (S/V) we also observed different results for the wild type cells from cold shock in comparison with those grown during 72h at 8°C, since the wild type strain maintained a similar S/V when was cultured at 30°C or 8°C but was higher in cells from cold shock experiments. Rod-shaped bacteria alter their width and length to achieve a S/V homeostasis [54] and changes in this parameter are observed in response to stress conditions [55], our observations in the wild type were in line with this idea and alteration in LPS or in different glycoforms balance lead to changes in this parameter as well as a shift in culture features.

**Fig 5:**
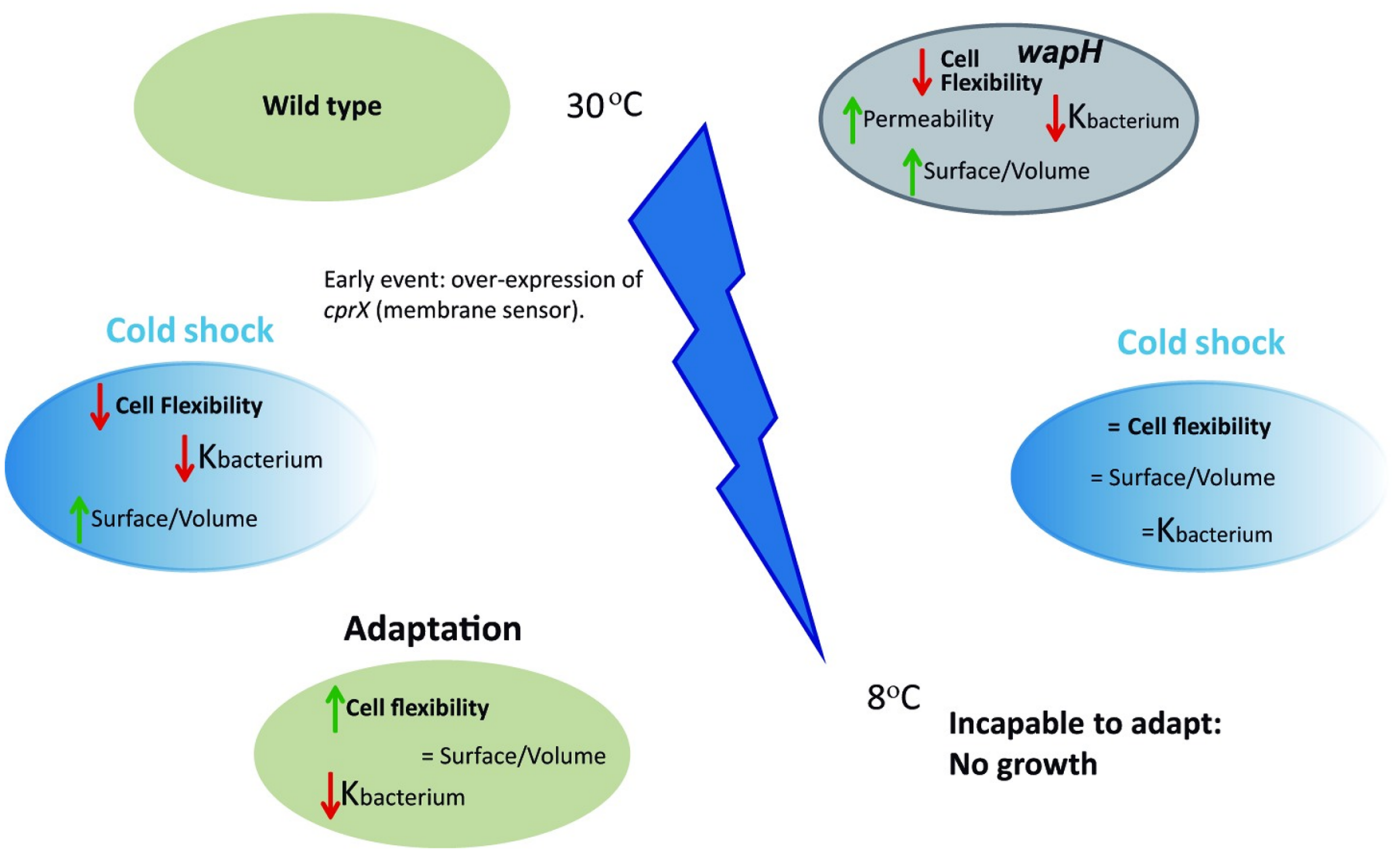
Proposed model to explain the effect of low temperatures in *P*.*extremaustralis* envelope characteristics and the impact of the *wapH* in cold adaptation.

Our study present evidences that LPS conservation is essential for development of *P*.*extremaustralis* under cold conditions and result a key structure for the maintenance of cell flexibility, turgor pressure and cell permeability as well as for S/V homeostasis. The capability of cells to modify physiological features is essential to reach an acclimation state (Fig 5). Our results showed a complex phenotype and along with other studies reveal multiple physiological adaptations for bacterial active growth under low temperatures.

## Supporting information

S1 Fig AFM representative measurements. A. Representative Force-distance curves obtained using MultiMode 8 with a Nanoscope V controller, Bruker in contact mode. B. Representative Image obtained with atomic force microscopy of *P*.*extremaustralis* grown at 30°C. Cells were imbibed in PEI as was described in Material and Methods.
(TIF)

S2 Fig Initial characterization of the *wapH* mutant strain. A. Growth at 8 in LB octanoate supplemented plates. Plates were incubated at 30°C during 24 h and at 8°C for a 1 week. B. Polyacrilamide analysis of LPS. Equal amount of Kdo was loaded and gel electrophoresis was performed. Bands were visualized using silver stain. Schematic representation of the structure of *P*.*aeruginosa* PAO1 LPS. Hexagonal forms represent hexose residues. Black arrows showed different glycoforms that can be synthase within a cell and grey arrow represents a glucose residue addition catalyzed by WapH. Long rectangle represents O-antigen. C. Attached biomass in mutant strain cultures in LB media supplemented with sodium octanoate. Cultures were incubated at 30°C. D. Expression of *wapH*, *mig14* and PE143B_0104935 measured by qPCR Real Time in cultures grown at 30°C. The results are expressed as fold change taking the wild type expression as 1. Results are shown as Mean±SD of at least 3 independent cultures for RNA extraction.
(TIF)
