# Supplementary figures and images for: Novel role of the LPS core glycosyltransferase WapH for cold adaptation in the Antarctic bacterium Pseudomonas extremaustralis
Source: PLoS One. 2018 Feb 7;13(2):e0192559. doi: 10.1371/journal.pone.0192559 (PMC5802925; doi:10.1371/journal.pone.0192559)

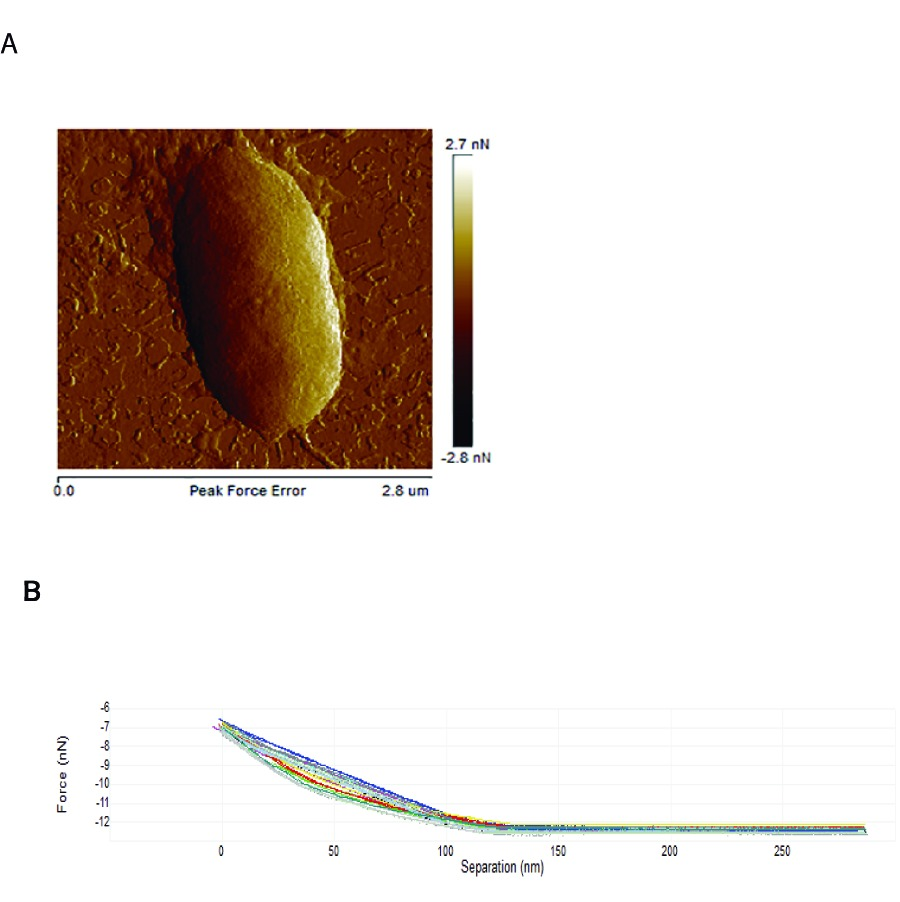

Supplement: S1 Fig — A. Representative Force-distance curves obtained using MultiMode 8 with a Nanoscope V controller, Bruker in contact mode. B. Representative Image obtained with atomic force microscopy of P.extremaustralis grown at 30°C. Cells were imbibed in PEI as was described in Material and Methods. (TIF) [file pone.0192559.s001.tif]

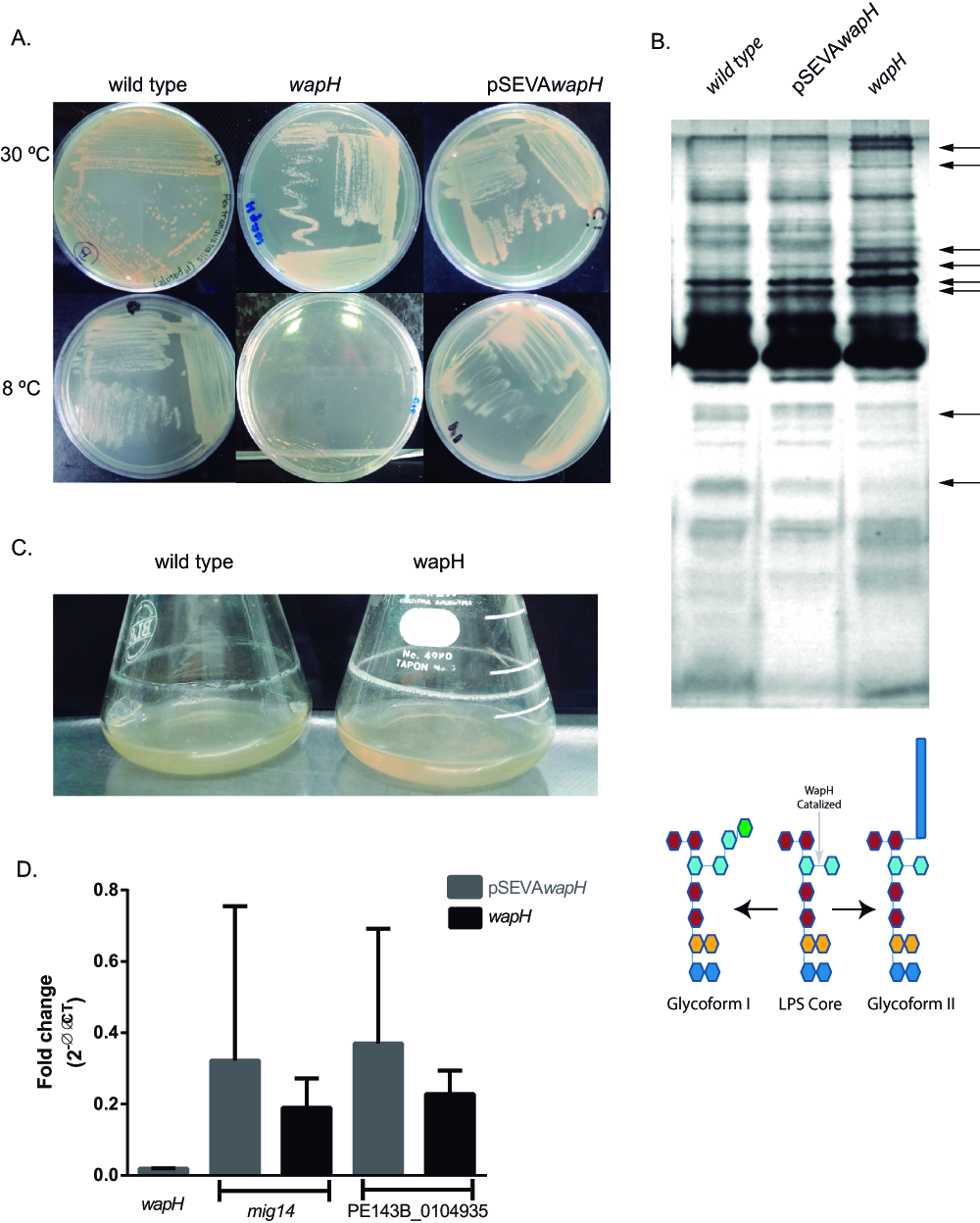

Supplement: S2 Fig — A. Growth at 8 in LB octanoate supplemented plates. Plates were incubated at 30°C during 24 h and at 8°C for a 1 week. B. Polyacrilamide analysis of LPS. Equal amount of Kdo was loaded and gel electrophoresis was performed. Bands were visualized using silver stain. Schematic representation of the structure of P.aeruginosa PAO1 LPS. Hexagonal forms represent hexose residues. Black arrows showed different glycoforms that can be synthase within a cell and grey arrow represents a glucose residue addition catalyzed by WapH. Long rectangle represents O-antigen. C. Attached biomass in mutant strain cultures in LB media supplemented with sodium octanoate. Cultures were incubated at 30°C. D. Expression of wapH, mig14 and PE143B_0104935 measured by qPCR Real Time in cultures grown at 30°C. The results are expressed as fold change taking the wild type expression as 1. Results are shown as Mean±SD of at least 3 independent cultures for RNA extraction. (TIF) [file pone.0192559.s002.tif]
